# P-706. Sputum Culture laboratory rejection based on patient demographics and co-morbidities

**DOI:** 10.1093/ofid/ofaf695.918

**Published:** 2026-01-11

**Authors:** William Leach

**Affiliations:** Geisinger, Danville, Pennsylvania

## Abstract

**Background:**

Sputum cultures often have high laboratory rejections rates. Improper collection techniques are common and add to rejection rates. Yet, sputum collection can significantly affect patient outcomes as the gram stan holds the potential to be the first actionable result in the setting of pneumonia. The aim of this study is to consider what patient factors are associated with rejected sputum samples to better understand how patients with pneumonia can receive improved care.

**Figure:**
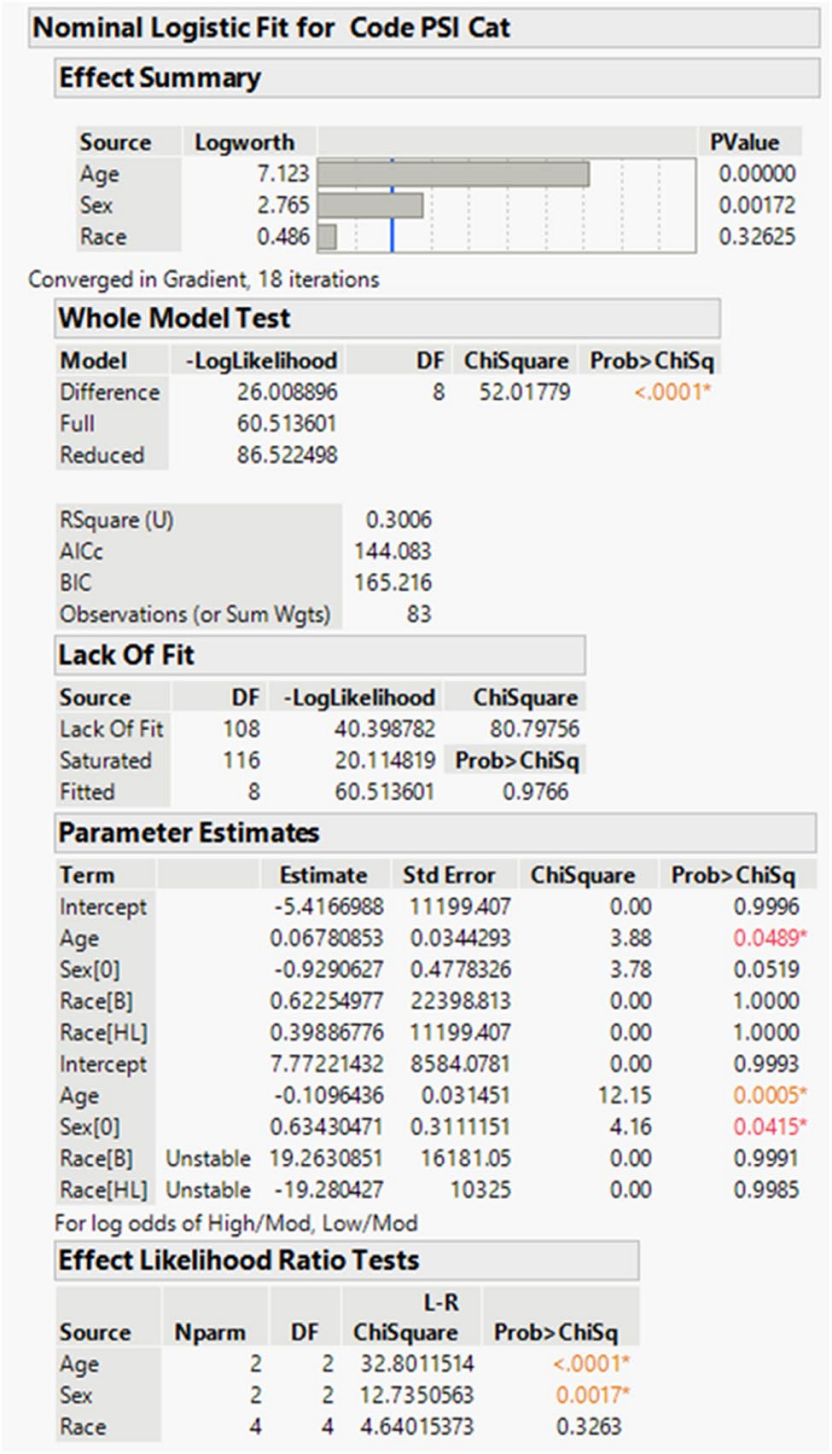
Logistical fit for Demographic comparison to sputum rejection

**Figure:**
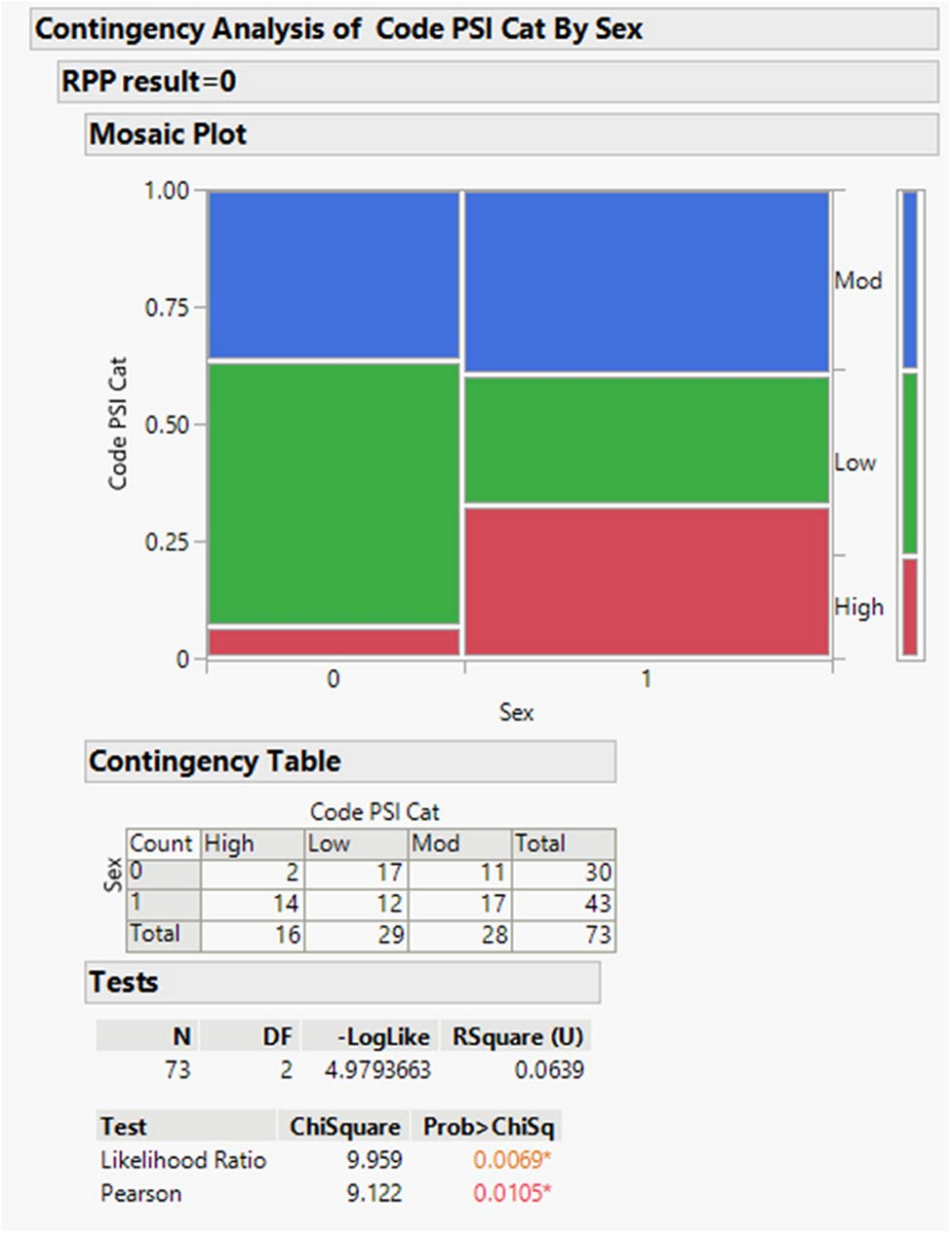
Gender significance regarding pneumonia severity index in rejected sputum samples

**Methods:**

In this single center retrospective study performed at Geisinger Medical Center, inpatient data from 2003 regarding rejected sputum cultures were analyzed. 130 patients over the age of 18 were selected for randomization which included: demographics, co-morbidities, laboratory results. The Pneumonia Severity Index (PSI) was calculated and categorized as low, moderate, and high severity, based on published findings. Patients intubated prior to or at the time of sampling or had a tracheostomy at time of sampling were rejected from analysis. The collected information was analyzed for differences in rejection rate based on patient demographics, comorbidities, laboratory results, and PSI category.

**Figure:**
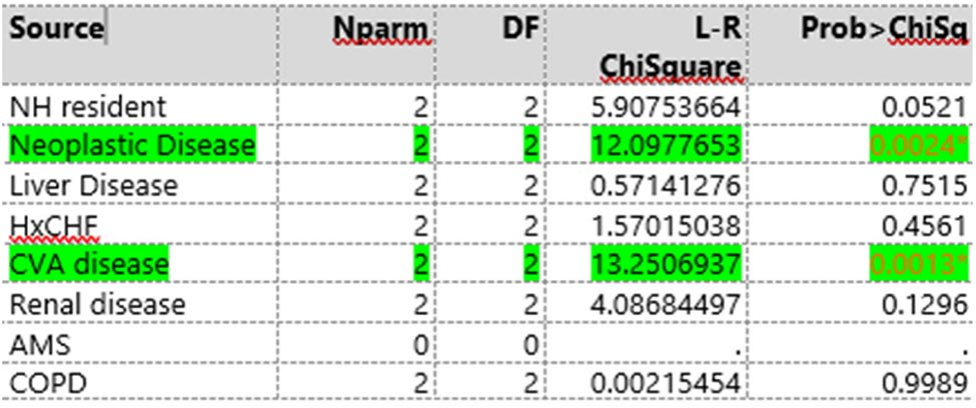
Male co-morbidities regarding sputum rejections

**Figure:**
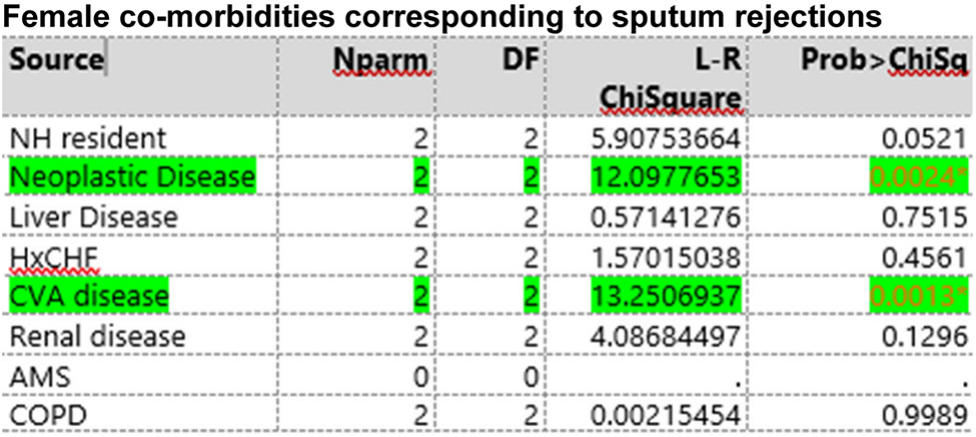
Female co-morbidities corresponding to sputum rejections

**Results:**

A total of 83 patients with rejected sputum samples underwent randomization. Patients had an average age of 70.0 years old (95% CI, 65.6 to 72.4), with 45% female (95% CI, 0.34 to 0.55) and 55% male patients (95% CI, 0.45-0.66). Demographic analysis via logistical regression compared to severity of PSI category showed a difference based on age (LR 32.8, P-value < 0.001) and gender (LR 12.7, P 0.0017. Regarding comorbidities for male patients, cerebral vascular accident (LR 13.3, P-value 0.0013), and presence of neoplastic disease (LR 12.1, P-value 0.0024) were significant determinants for rejection. In female patients, renal disease (LR 6.9, P-value 0.0324), heart failure (LR 6.23, P-value 0.044), and neoplastic disease (LR 19.3, P-value 0.0001) were significant factors for sputum.

**Conclusion:**

Regarding sputum rejection from laboratory analysis based on sample quality, new associations have been identified. Future intervention based on the identified associations with population-specific education or pre-analytic options may improve the rate of sputum acceptance for future interventions.

**Disclosures:**

All Authors: No reported disclosures

